# On-Target Low-Density Lipoprotein Cholesterol in Adults with Diabetes Not at High Cardiovascular Disease Risk Predicts Greater Mortality, Independent of Early Deaths or Frailty

**DOI:** 10.3390/jcm13247667

**Published:** 2024-12-16

**Authors:** Bruna C. Chwal, Rodrigo C. P. dos Reis, Maria I. Schmidt, Antonio L. P. Ribeiro, Sandhi M. Barreto, Rosane H. Griep, Paulo A. Lotufo, Bruce B. Duncan

**Affiliations:** 1 Programa de Pós-Graduação em Epidemiologia, Universidade Federal do Rio Grande do Sul, Porto Alegre 90035-003, Brazil; brunacristine.chwal@gmail.com (B.C.C.); rodrigocpdosreis@gmail.com (R.C.P.d.R.); maria.schmidt@ufrgs.br (M.I.S.); 2 Departamento de Estatística, Universidade Federal do Rio Grande do Sul, Porto Alegre 91509-900, Brazil; 3 Hospital de Clínicas de Porto Alegre, Porto Alegre 90035-903, Brazil; 4 Faculdade de Medicina e Hospital das Clínicas/EBSERH, Universidade Federal de Minas Gerais (UFMG), Belo Horizonte 30130-100, Brazil; 5 Laboratório de Educação em Ambiente e Saúde, Instituto Oswaldo Cruz, Fundação Oswaldo Cruz, Rio de Janeiro 21040-900, Brazil; 6 Center for Clinical and Epidemiologic Research, University of São Paulo, Sao Paulo 05508-000, Brazil

**Keywords:** diabetes mellitus, type 2, cholesterol, LDL, mortality, cardiovascular risk

## Abstract

**Background/Objectives:** Lowering low-density lipoprotein cholesterol (LDL-C) to <70 mg/dL is recommended for most patients with diabetes. However, clinical trials investigating subjects with diabetes who are not at high cardiovascular risk are inconclusive regarding the all-cause mortality benefit of the current target, and real-world studies suggest greater mortality. We aimed to assess the all-cause mortality at different LDL-C levels among subjects with diabetes not at high risk and to examine the potential roles of early deaths and frailty for this greater mortality. **Methods:** We followed 2098 such participants of the ELSA-Brasil cohort between 2008 and 2019. **Results:** Over 10.3 (1.4) years of follow-up, 204 (9.7%) individuals died. In the proportional hazards models, participants with LDL-C values < 100 mg/dL and <70 mg/dL had greater adjusted mortality compared to those with LDL-C 100–129 mg/dL (HR = 1.67; 95%CI 1.21–2.30 and HR = 2.27; 95%CI 1.51–3.41, respectively). Increased risk when LDL-C was <100 mg/dL was higher in those >60 years (HR = 2.12; 95%CI 1.35–3.34) and greatest for deaths due to cancer (HR = 2.55; 95%CI 1.10–5.91). Further analyses for those with LDL-C < 100 mg/dL that excluded early deaths and adjusted for the frailty phenotype (HR = 2.01; 1.19–3.41) or frailty index (HR = 1.92; 1.17–3.16) did not materially alter the results. The risk of death across the spectrum of LDL-C was U-shaped, with a nadir at 112.2 mg/dL. **Conclusions:** The higher risk of all-cause mortality in these subjects with LDL-C within currently recommended levels was not explained by early deaths or frailty. Given the recent decline in cardiovascular mortality and the increased risk of cancer and infections in persons with diabetes, the clinical significance of low LDL-C in diabetes requires reconsideration and the definition of LDL-C treatment targets in diabetes warrants further trial evaluation.

## 1. Introduction

The benefit of lipid-lowering therapy to prevent cardiovascular disease (CVD) among individuals with established cardiovascular disease is well established by multiple clinical trials. However, trial results among those with diabetes who are not at high CVD risk are less consistent [1,2].

In addition, real-world observational studies have found that low levels of low-density lipoprotein cholesterol (LDL-C) increase the risk of death in subjects with diabetes [3,4,5]. LDL-C may be a marker of greater mortality risk due to frailty, malnutrition, or other causes of impending death. Alternatively, low levels of LDL-C may themselves cause either the onset or worsening of some infections [4] and cancer [6]. The importance of considering this latter hypothesis is rising since the decline in cardiovascular deaths has changed the mortality profile of diabetes, with a greater fraction now resulting from cancer and dementia, as well as liver and infectious diseases. In England in 2018, only approximately one-quarter of deaths in patients with diabetes were due to vascular causes, which was less than those due to cancer [7]. Additionally, much of the CVD mortality is clustered in a relatively small fraction of patients with identifiably high CVD risk.

Despite inconsistency in the trial and observational study results, most authorities recommend tight lipid-lowering treatment targets for primary prevention in most subjects with diabetes. The European Society of Cardiology (ESC) recommends lipid-lowering to an LDL-C goal of <55 mg/dL for all subjects with very high risk, <70 mg/dL for those with high risk, and <100 mg/dL for those with moderate risk [8]. The American Diabetes Association (ADA) recommends lipid-lowering to an LDL-C goal of <70 mg/dL (1.81 mmol/L) for all subjects with diabetes aged 40–75 without known atherosclerotic CVD but with at least one additional CVD risk factor [9]. Other major society guidelines have lowered targets in recent years to levels similar to those of the ESC and ADA [10,11]. The greater mortality described in observational studies was not addressed in these guidelines.

We aimed to evaluate the all-cause mortality related to LDL-C levels in adults with known diabetes but not at high risk for CVD who were participating in the ELSA-Brasil study. This large contemporary cohort of middle-aged and elderly Brazilians also allowed the investigation of early deaths or frailty as explanations for the association.

## 2. Materials and Methods

### 2.1. Study Population and Design

A total of 15,105 civil servants aged 35 to 74 at higher education and research institutions in six Brazilian capital cities from 2008-10 (Visit 1) were enrolled in the ELSA-Brasil study [12]. Following this, two additional onsite visits (2012–2014 and 2017–2019) were conducted, and an annual telephone surveillance was implemented to detect deaths. Deaths were confirmed through hospital records, autopsies, and death certificates. For this study, we included only participants with self-reported diabetes or pharmacologic treatment for diabetes, considering their baseline to be the visit at which diabetes was ascertained.

### 2.2. Ethical Approval and Consent to Participate

ELSA-Brasil study was approved by the research ethics committees of participating institutions. All participants gave their written informed consent.

### 2.3. Measurements

Data collection occurred in clinical research centers during the three visits as previously described [13,14,15]. We conducted standardized interviews and clinical assessments, in addition to collecting blood and urine samples after an overnight (>8 h) fast at all visits. Samples obtained were frozen and shipped to a central laboratory for determination. The study collected baseline data on age, sex, self-reported race/ethnicity group (white, mixed, black, Asian, and Indigenous), smoking, medical history of a diabetes diagnosis, and use of anti-diabetic and cholesterol-lowering medications through interviews. Systolic and diastolic blood pressures were calculated as the average of the last two of three measurements taken. Weight, waist circumference, and height were measured while fasting and with an empty bladder. The estimated glomerular filtration rate (eGFR) was calculated using the CKD-Epi equation without adjustment for race. We considered participants to have previously known diabetes at Visits 1–3 when they reported having been previously diagnosed by a physician and were using anti-diabetic medication. We estimated ten-year risk of a major cardiovascular event (myocardial infarction, stroke, or cardiovascular death) based on age, sex, smoking, systolic blood pressure, and total cholesterol according to the WHO Risk Chart Working Group table for the Tropical Latin America region [16]. When >20%, we categorized CVD risk as high.

We assessed frailty using the frailty phenotype and the frailty index. The frailty phenotype was based on unintentional weight loss, self-reported exhaustion, low energy expenditure, slow gait speed, and weak grip strength, with definitions adapted from the Cardiovascular Health Study (Table S1) [17,18]. The presence of three or more of the above five factors characterized frailty and of one or two factors a pre-frail state. The frailty index, as previously developed in ELSA-Brasil (Table S2) [17,19,20,21], was based on the cumulative deficit approach [22], with frailty defined categorically as a frailty index ≥0.25. These frailty measures were applied as a covariate only in participants with follow-up data beyond ELSA Visit 3, during which most of the variables needed to produce the index were obtained.

### 2.4. Definition of LDL-C Target Levels

Our analyses considered LDL-C targets below 100 mg/dL (2.59 mmol/L) and 70 mg/dL (1.81 mmol/L). We chose 100 mg/dL for the target cutoff as preliminary analyses suggested that this point was close to the mortality nadir found for LDL-C, has been proposed as a goal in guidelines, and provides a more conservative goal that is perhaps more appropriate for use in many low- and middle-income country settings in which resources available for health care and access to more potent medications, such as SGLT2 inhibitors, GLP-1 agonists, and stronger lipid-lowering drugs, are more limited. We chose 70 mg/dL for sensitivity analyses as this level is frequently suggested in guidelines as a goal in CVD prevention for those with diabetes with greater but not high CVD risk. We assessed target achievement based on values obtained at the visit during which diabetes was first ascertained.

As guidelines have lowered LDL-C targets for those in primary prevention with evidence of greater CVD risk, we calculated the fraction of our sample with at least one other CVD risk (hypertension, glycated hemoglobin > 8%, smoking, obesity, duration of diabetes of 10 or more years, or chronic kidney disease, including an evaluation for albuminuria).

### 2.5. Outcomes

Participants with known diabetes at Visit 1 were followed until 1 October 2023. Those with diabetes ascertained at Visits 2 and 3 established their baseline at the visit of ascertainment, with follow-up beginning at that point (Figure S1). Although ELSA-Brasil is still finalizing the adjudication of the causes of participants’ deaths, the majority of the death causes have already been centrally adjudicated.

### 2.6. Statistical Analyses

Categorical variables were described as frequencies and percentages and continuous ones as means and standard deviations (SD). The chi-square test was used to statistically test crude associations between target achievement and mortality.

Cox proportional hazards models were performed, adjusting for age, sex, educational achievement, waist–hip ratio, HbA1c, systolic blood pressure, statin use, private health insurance, albumin–creatinine ratio, eGFR, and smoking, to evaluate the association between LDL-C control and all-cause mortality, stratified by age, sex, statin use, cause of death, and frailty. Similar models were used to investigate associations with cause-specific mortality. Sensitivity analyses were performed by excluding deaths during the first five years of follow-up and adjusting for frailty. Primary analyses categorically investigated the level of control, comparing deaths in participants with values below the target cutoff to those in the 100–129 mg/dL range. To capture nonlinear relationships of the LDL-C level with death, we additionally determined the risk of death at several levels of LDL-C. We investigated the association when LDL-C was expressed as a continuous variable via Cox proportional hazards models with restricted cubic splines. We chose 3 knots in these, placing them at the 10th, 50th, and 90th LDL-C percentiles [23].

All data analyses were performed using the software R [version 1.3. 1056, © 2024-2020], R Foundation for Statistical Computing (Vienna, Austria).

## 3. Results

Of the 15,105 participants at baseline, 2540 (16.8%) reported a diagnosis of diabetes or use of medication to treat diabetes during the three study visits: 1279 at baseline, 530 at Visit 2, and 731 at Visit 3. After excluding 117 participants with missing or incomplete data for LDL-C and covariate levels and 338 with a history of atherosclerotic CVD or high CVD risk, our final sample comprised 2098 participants (Figure S1).

The mean (SD) age at baseline was 55.6 (8.6). Additional baseline characteristics of the study sample are presented in Table 1. A total of 1111 (53.0%) participants were men, 309 (14.7%) were older than 64 at baseline, and 929 (44.3%) self-declared as white race-color. A total of 1157 (55.1%) reported less than university education, 1234 (58.8%) had private health insurance, 756 (36.0%) had income less than four Brazilian minimum wages, 1821 (86.8%) were not currently smoking, and 781 (39.5%) were receiving statins and 160 (9.0%) either GLP-1 agonists or SGLT2 inhibitors. A total of 1735 (82.7%) had BMI ≥ 25 kg/m^2^, and 1712 (82.8%) had one or more additional CVD risk factors (hypertension, glycated hemoglobin >8%, duration diabetes > 10 years, age > 60, obesity or overweight, smoking, and chronic kidney disease, including the presence of albuminuria). Additionally, 102 (9.6%) were frail according to the frailty index and 90 (9.6%) based on the definition of the physical frailty phenotype. Those deemed frail had a considerably greater risk of death compared to the remaining ones. Concerning LDL-C, 732 (34.9%) had values <100 mg/dL, including 185 (8.9%) with values <70 mg/dL.

During an average of 10.3 (1.4) years, 204 (9.7%) participants died. Of the 144 whose cause of death had been adjudicated, 53 (36.8%) died of cardiovascular disease, 48 (33.3%) of cancer, 10 of digestive diseases, 8 of chronic kidney disease, 7 of injuries, and 18 of other causes (chronic respiratory, blood, neurological, mental, and musculoskeletal diseases) (Table S3).

The crude and adjusted mortality were highest in the lowest LDL-C strata, were least between 100 and 160 mg/dL, and increased after that. Individuals with LDL-C < 100 mg/dL, compared to those with LDL-C between 100 and 129 mg/dL, had greater mortality in both crude (HR = 1.66; 95%CI 1.29–2.14) and adjusted (HR = 1.67; 95%CI 1.21–2.30) analyses. The risk was especially high in those with LDL-C values < 70 mg/dL (adjusted HR = 2.27; 95%CI 1.51–3.41) (Table 2).

Among those with LDL-C < 100 mg/dL, the risk was somewhat higher in those aged 60 or older (HR = 2.12; 95%CI 1.35–3.34, *p* ≤ 0.001 for interaction) and those who did not use statins (HR = 1.81; 95%CI 1.14–2.68, *p* = 0.123 for interaction). Investigating specific causes of death, we found a greater risk of dying from cancer (adjusted HR = 2.55; 95%CI 1.10–5.91) (Table 2).

To evaluate non-causal reasons to explain these findings, we first excluded those who had died during the first five years of their follow-up (*n* = 50, 20.7% of deaths). The risk associated with being below 100 mg/dL remained unchanged (HR = 1.60, 95%CI 1.19, 2.16) (Table 2).

To investigate this further, we evaluated the potential role of frailty in these associations. In these analyses, we could only consider participants followed beyond the third clinic visit (*n* = 941 for the biological phenotype and 1067 for the index), at which most of the items of our frailty constructs had been ascertained (Figure S1).

A comparison of mean baseline LDL-C levels between robust and frail individuals, whether the latter was assessed considering the biological phenotype or the frailty index, revealed similar values (116 mg/dL vs. 118 mg/dL and 117 mg/dL, respectively).

In regression analyses additionally adjusting for frailty, associations for those with LDL-C <100 mg/dL remained similar to those found in the overall sample (for phenotype: HR = 2.05, 95%CI 1.21–3.47 and for the index: HR = 1.94, 95%CI 1.19–3.17). Additionally, analyses considering only deaths more than five years from diabetes ascertainment, when also adjusted for frailty, produced associations of similar size (HR = 2.01; 95%CI 1.19–3.14 for the frailty phenotype and HR = 1.92, 95%CI 1.17–3.16 for the frailty index) (Table 3).

**Figure 1:**
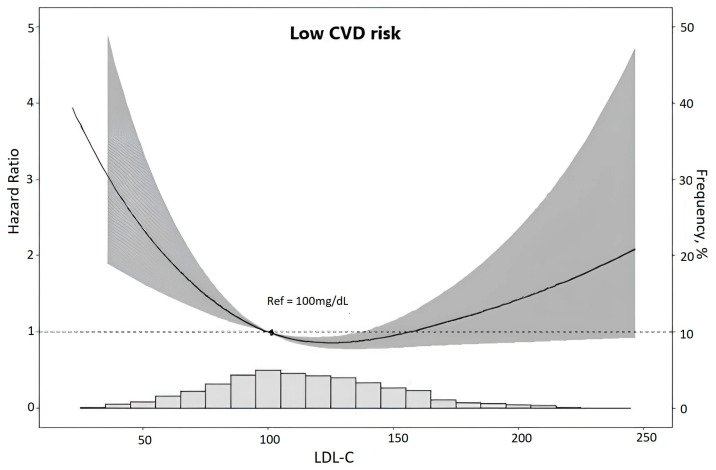
Risk of death (gray area represents the zone of 95% confidence) according to LDL-cholesterol levels in low CVD risk participants. Associations were obtained through restricted cubic spline analyses using Cox proportional hazards models adjusted for age, sex, race, per capita income, private health insurance, smoke, statin use, waist–hip ratio, HbA1c, albumin–creatinine ratio, estimated glomerular filtration rate, and systolic blood pressure. The y-axis to the left indicates the hazard ratio of death. The y-axis to the right indicates the relative frequency (%) of LDL-cholesterol levels displayed in the superimposed histogram at the bottom of the figure.

Finally, to describe the mortality risk across the complete spectrum of LDL-C, we plotted the adjusted risk of death according to continuously expressed LDL-C in the whole sample (Figure 1). In the resultant U-shaped curve, the greatest mortality was present at the lowest LDL-C levels, with a nadir at 112.2 mg/dL and a trend of increased risk at the highest values.

## 4. Discussion

Among middle-aged and elderly Brazilian adults with known diabetes not presenting high CVD risk, mortality was 67% greater among those having an LDL-C < 100 mg/dL compared to those with levels between 100 and 130 mg/dL. The risk was 127% greater when LDL-C was <70 mg/dL. An increased mortality risk for those with LDL-C within clinical targets persisted after adjustment for multiple potential confounders. It was not explained by deaths occurring earlier during the follow-up or by frailty. The relative risk at or below the target LDL-C levels was greatest in those aged 60 or more. Additionally, those with LDL-C < 100 mg/dL had more deaths related to cancer, numerically fewer CVD deaths, and a somewhat greater number of deaths from other causes.

Diabetes has been previously considered a “CVD risk equivalent”, although multiple studies have now shown that this is not the case [24]. For example, among participants with diabetes ascertained by HbA1c in a representative sample of Brazilian adults, only 17.8% had known cardiovascular disease, and only an additional 3.4% had a >20% 10-year probability of a major CVD event based on the WHO cardiovascular risk charts [25]. Thus, we focused our analyses specifically on those adults with diabetes who are not at high CVD risk, who constitute the majority of subjects, and for whom the question of how low to aim LDL-C reduction is most relevant.

The trial findings in individuals with diabetes have shown that the benefit of lipid-lowering is open to question. First, neither the meta-analysis nor the three trials reported in the meta-analyses have shown a statistically significant reduction in all-cause mortality in diabetic individuals without known cardiovascular disease [1,2]. Second, these trials have included few elderly subjects, raising a question of their capacity to generalize their findings to this large segment of the diabetic population [26]. Third, the CVD primary prevention trial in diabetes that reported the greatest reduction in all-cause deaths, MEGA, had an on-treatment mean LDL-C of approximately 127 mg/dL [1,2,27], far from the current recommendations. In a more recent lipid-lowering trial with bempedoic acid, participants with diabetes without a previous history of CVD had a 17% reduction in all-cause mortality (HR 0.83; 95%CI 0.53–0.92). However, this trial does not validate the currently recommended lipid-lowering targets, as those with diabetes in the intervention arm did not achieve a mean LDL-C on treatment below 95 mg/dL [28].

Additionally, the selective recruitment of patients for these primary prevention trials raises issues of their generalizability. In general, participants with diabetes, despite not necessarily being at high risk, on average, had a greater risk of dying from CVD than those with diabetes, either due to explicit entrance criteria or simply from their recruitment in settings of greater CVD risk. This selective recruitment would produce relatively more CVD deaths (against which a lower LDL-C protects) and fewer non-CVD deaths, particularly from cancer (where low LDL-C levels appeared harmful in our sample and in others) than in those with diabetes but without high CVD risk overall. The fact that some of these trials were conducted decades ago accentuates this problem, as cardiovascular deaths relative to those from cancer and other causes have decreased over time [7,29]. The recent bempedoic acid trial also likely presented this problem of selective recruitment.

Given the uncertainty in trial results, examining the findings of observational studies addressing the relationship between LDL-C levels and all-cause mortality becomes relevant. Previous studies in general populations have shown higher all-cause mortality within the recommended LDL-C range [30], particularly so in older adults [31,32]. Observational studies not specifically investigating diabetes have suggested a potential U-shaped or reverse J-shaped curve, indicating that not only high but also low levels of LDL-C may be associated with an increased risk of all-cause mortality [33,34].

Our findings of greater mortality at low LDL-C levels in individuals with diabetes confirm those of other cohort studies of participants with diabetes. The TRIAD study, a multicenter, prospective, observational study of 8733 patients with diabetes treated in managed care in the U.S., found, in multiply-adjusted analyses, that those without dyslipidemia had a 24% higher all-cause mortality [3]. An LDL-C between 100 and 129 mg/dL was associated with the lowest mortality in a large hospital outpatient sample with diabetes in Taiwan [35]. Among subjects followed for death up to 2015 in nationally representative NHANES 1999–2014 surveys, those with diabetes also had minimum mortality in this range, while those with LDL-C < 70 mg/dL had a 55% (HR = 1.55; 95%CI 1.10–2.19) greater risk of death from any cause [4].

The possibility that baseline LDL-C is merely a marker of diseases soon to cause death has been raised to explain why trials show protection with lipid-lowering and real-world studies show risk at lower LDL-C levels. To investigate this hypothesis, we explored two potential non-causal reasons for the greater mortality found: a low LDL-C was a manifestation of impending death or a characteristic of frailty. The minimal change in risk resulting from excluding deaths during the first five years of follow-up makes the former an unlikely explanation. The similar levels of LDL-C in frail and robust individuals in our cohort raise doubt about the generalizability of the hospital-based findings [36] of an association between low LDL-C and frailty in general populations. These similar levels and our findings of little change in risk of death after adjusting for the frailty index or frailty phenotype make frailty also an unlikely explanation. The fact that low LDL-C correlates with hypoalbuminemia, a known marker of increased risk of death, has also been used to explain the association of low LDL-C with greater mortality. While we cannot investigate this hypothesis in our sample, a recent study following 46,675 patients with diabetes in Taiwan from 2003 to 2018 showed that low LDL-C increased the risk of death independent of the level of albumin [5].

A possible benefit of lipid-lowering in general, but not to the LDL-C levels currently recommended by the ADA, is supported by the meta-regression analyses of trial results that were not restricted to those with diabetes. In this meta-regression of mortality by pre-trial LDL-C level, an overall benefit of lipid-lowering was present. However, the reduction in mortality decreased by 9% for each 40 mg/dL decrease in baseline LDL-C as it went from ~200 mg/dL to ~100 mg/dL. Furthermore, at 100 mg/dL, no reduction in all-cause mortality was present [37]. Two other studies investigating meta-regression in lipid-lowering trials showed that while statin-induced lipid-lowering was not associated with cancer incidence, the lower the level reached in the lipid-lowering arms of the trials included, the higher the cancer incidence. Cancer incidence in patients with an achieved mean LDL-C of ~90 mg/dL was more than double that in patients with an achieved mean of ~140 mg/dL [38,39]. Of note, the lowest achieved LDL-C mean in the studies was higher than the below 70 mg/dL goal currently recommended for most with diabetes by the current guidelines. An observational comparison of 21,294 matched pairs of U.S. veterans with diabetes and without cardiovascular disease treated with statins achieving mean LDL-C during follow-up of <70 mg/dL vs. 70–100 mg/dL demonstrated no benefit with stricter control (OR = 0.97; 0.92–1.02). Notably, both groups investigated were in the range of greater risk found in the observational studies [40]. A meta-analysis demonstrating statins to be protective against cancer [41] may not be generalizable to treatments achieving values considerably below 100 mg/dL, as the LDL-C level achieved was not presented and may have been considerably higher than those in the current guidelines.

Mendelian randomization studies, on balance, support a causal role for low LDL-C. Though an initial 2011 Mendelian randomization study [42] found no increased cancer risk with low LDL-C levels, more recent ones, incorporating a greater number of genes, have found purportedly causal associations for endometrial cancer [43], hepatocellular carcinoma [44], and lung cancer [45].

However, to our knowledge, mechanistic explanations for a causal pathway from low levels of LDL-C to cancer are limited. One suggested explanation is that a low level of LDL-C significantly affects antitumor immunity, increasing cancer risk [6]. However, others have proposed mechanisms for the opposite, for higher levels of LDL-C to cause cancer [46].

Pathways that are apparently non-causal, supported by empirical findings, have been suggested to explain the association of low LDL-C with cancer. Low LDL-C may reflect abnormal cholesterol biosynthesis resulting from inflammation and oxidative stress secondary to chronic hyperglycemia and reduced insulin secretion. The latter, in parallel, may activate the RAS signaling pathway, producing an increased cancer risk [47]. In fact, in the setting of low LDL-C and albuminuria, statins were associated with lower mortality [48], though perhaps partly because low levels in patients receiving statins were statin-induced rather than by some pathophysiologic mechanism.

An increased risk of infection is another postulated means by which a low LDL-C could lead to a greater risk of death. LDL particles have been found to bind to and inactivate a broad range of microorganisms and their toxic products [4]. In an extensive study of dialysis patients, having a low LDL-C increased the risk of death by infection [49]. In a representative study of patients in the United Kingdom, infection was the third leading cause of death in diabetes [50]. As infection is a frequent immediate or contributing cause of death for a variety of other underlying causes, a greater risk of infection due to low LDL-C could have a meaningful impact on all-cause mortality in diabetes.

In sum, previous studies have demonstrated that diabetes is not, by definition, a “CVD risk equivalent” high-risk state. Our empirical findings, supported by those of other studies [3,4,35], suggest the need to consider different approaches for those with diabetes who are not at a high CVD risk. We may go beyond this and suggest just the opposite—that diabetes, given its increased risk for cancer and infections, is a high-risk setting for those having low LDL-C. Perhaps surprisingly, this affirmation is less discordant from clinical trials than one might imagine. A recent systematic review of lipid-lowering trials, not limited to diabetes, while not finding harm from lipid-lowering to the level of current guidelines, also found no additional benefit of lowering LDL-C below 130 mg/dL [51]. The existence of newer, more potent, lipid-lowering medications makes this issue all the more important.

Potential limitations of our study should be acknowledged. Our cohort findings, by design, present more risks to their validity when compared to those of randomized trials. However, randomized trials to date, as opposed to our cohort sample, have limited generalizability to the spectrum of low LDL-C values that constitute the goals of current guidelines. The generalizability of our findings can also be questioned. However, while our Brazilian subjects’ epidemiological profile, healthcare access, and cultural factors could influence outcomes in ways that may not be directly applicable to populations elsewhere, we believe that this biologically based association of low LDL-C with mortality should be generalizable to most populations of type 2 diabetes around the world. The absence of data on retinopathy and other diabetes-related complications associated with mortality in the ELSA-Brasil study restricts our ability to adjust for these variables. However, given that we have already accounted for cardiovascular and renal disease risk factors, further adjustments for retinopathy and other complications would likely have had minimal impact on the hazard ratios. We did not analyze associations in adults with diabetes who were at high CVD risk, as we lacked the statistical power to address the issue in this setting. Thus, our findings are not generalizable to situations with high CVD risk. Finally, the small number of deaths and the limited statistical power to analyze cause-specific mortality mean that our cause-specific findings should be considered preliminary.

Our study’s strengths include its contemporary sample of participants residing in multiple locations across Brazil, its careful and extensive collection of data on risk factors and covariates, its standardized and centralized laboratory measurements, and the broad characterization of ELSA-Brasil participants that permits the investigation of potential non-causal reasons for this association.

## 5. Conclusions

In conclusion, consistent with the previous observational studies, we found greater mortality at lower LDL-C levels for adults with diabetes but without high CVD risk. Despite consistent observational findings and meta-regression of trial findings showing decreased mortality benefit and increased cancer risk with decreasing on-treatment LDL-C values, recommendations of various societies have progressively called for increasingly lower lipid targets. Taken together and considering the recent decline in cardiovascular mortality and the increased cancer and infectious disease risk in persons with diabetes, overall findings call for the reconsideration of the meaning of a low LDL-C value in diabetes and additional clinical trials to best define the ideal LDL-C targets for lipid-lowering in patients with diabetes not at high CVD risk. Until new trial findings on all-cause mortality are available, emphasis in recommendations to achieve LDL-C targets in guidelines should be tempered, given conflicting evidence of the benefit of lipid-lowering beyond 100 mg/dL. 

## Figures and Tables

**Table 1:** Baseline socio-demographic and clinical characteristics of 2098 adults with known diabetes. ELSA-Brasil, 2008 to 2023.

	All	Alive	Died	*p* *
Characteristic	*n* = 2098 *n* (%)	*n* = 1894 (90.2%) *n* (%)	*n* = 204 (9.72%) *n* (%)	
Sex				<0.001
Male	1111 (53.0)	972 (87.5)	139 (12.5)	
Female	987 (47.0)	922 (93.4)	65 (6.59)	
Age (years)				<0.001
44 to 54	1022 (48.7)	975 (95.4)	47 (4.60)	
55 to 64	767 (36.6)	672 (87.5)	95 (12.4)	
>64	309 (14.7)	247 (79.9)	62 (20.1)	
Ethnicity				0.536
Black	435 (20.7)	385 (88.5)	50 (11.5)	
* Pardo*	600 (28.6)	539 (89.8)	61 (10.2)	
White	929 (44.3)	849 (91.4)	80 (8.61)	
Asian	110 (5.24)	99 (90.0)	11 (10.0)	
Indigenous	24 (1.14)	22 (91.7)	2 (8.33)	
Education				<0.001
Less than university	1157 (55.1)	1008 (87.1)	149 (12.9)	
University	941 (44.9)	886 (94.2)	55 (5.8)	
Private health insurance				<0.001
Yes	1234 (58.8)	1138 (92.2)	96 (7.78)	
No	864 (41.2)	756 (87.5)	108 (12.5)	
Income (minimum wages) **				0.040
<4	756 (36.0)	685 (90.6)	71 (9.39)	
4 to 7	690 (32.9)	608 (88.1)	82 (11.9)	
≥8	652 (31.1)	601 (92.2)	51 (7.82)	
Current smoking				0.153
Yes	277 (13.2)	243 (87.7)	34 (12.3)	
No	1821 (86.8)	1651 (90.7)	170 (9.34)	
Statin use				0.012
Yes	781 (39.5)	701 (89.8)	80 (10.2)	
No	1195 (60.5)	1112 (93.1)	83 (7.0)	
GLP-1RA or SGLT2i use at baseline				0.202
Yes	160 (9.0)	157 (98.1)	3 (1.9)	
No	1622 (91.0)	1552 (95.7)	70 (4.3)	
BMI (kg/m^2^)				0.085
<25	363 (17.3)	317 (87.3)	46 (12.7)	
25–29.9	855 (40.8)	768 (89.8)	87 (10.2)	
30–34.9	598 (28.5)	551 (92.1)	47 (7.86)	
≥35	282 (13.4)	258 (91.5)	24 (8.51)	
Waist–hip ratio M (SD)	0.96 (0.08)	0.96 (0.08)	0.98 (0.07)	0.004
Alb–Cr ratio (mg/g) M (SD)	38.0 (236)	19.9 (86.4)	206 (688)	<0.001
eGFR (mL/min per 1.73 m^2^) M (SD)	82.4 (16.4)	83.3 (15.5)	73.8 (21.1)	<0.001
Having additional CVD risk factors ***				<0.001
None	355 (17.2)	353 (99.4)	2 (0.6)	
One or more	1712 (82.8)	1513 (88.4)	199 (11.6)	
Frailty Index (*n* = 1067)				0.008
Robust	965 (90.4)	926 (96.0)	39 (4.0)	
Frail	102 (9.6)	94 (92.2)	8 (7.8)	
Physical frailty phenotype (*n* = 941)				0.001
Robust	553 (58.8)	534 (96.6)	19 (3.4)	
Pre-frail	298 (31.7)	289 (97.0)	9 (3.0)	
Frail	90 (9.6)	79 (87.8)	11 (12.2)	
LDL-C strata (mg/dL)				<0.001
<55	52 (2.5)	41 (78.8)	11 (21.2)	
55 to 69	133 (6.3)	107 (80.5)	26 (19.5)	
70 to 99	547 (26.1)	488 (89.2)	59 (10.8)	
100 to 129	661 (31.5)	613 (92.7)	48 (7.3)	
130 to 159	455 (21.7)	420 (92.3)	35 (7.7)	
≥160	250 (11.9)	225 (90.0)	25 (10.0)	

*n* (%) unless otherwise indicated; M (SD) = Mean (Standard Deviation); Alb–Cr ratio = Albumin–Creatinine ratio; eGFR = Estimated glomerular filtration rate; BMI = body mass index; LDL-C = low-density lipoprotein cholesterol; CVD = cardiovascular disease; GLP-1RA = GLP-1 receptor agonist; SGLT2i = sodium glucose cotransport 2 inhibitor. * Chi-square test was used to determine statistical significance of categorical variables and ANOVA was used to determine the difference in variable level or frequency of continuous variables. ** The monthly minimum wage was BRL 986.00 at the study midpoint. Numbers are multiples of this wage. *** Additional CVD risk factors: presence of hypertension, glycated hemoglobin > 8%, duration of diabetes > 10 years, age > 60 years, presence of obesity/overweight, currently smoking, and chronic kidney disease (estimated glomerular filtration [eGFR] < 60 mL/min/1.73 m^2^ or the presence of micro- or macroalbuminuria).

**Table 2:** Crude and adjusted * risks of death according to LDL-C values and levels compatible with clinical recommendations for individuals with known diabetes who are not at high CVD risk, overall, by specific demographic and clinical characteristics, and after exclusion of first years of follow-up. ELSA-Brasil, 2008 to 2023. *n* = 2098.

	Death		
	Crude	Adjusted *	*p*
Characteristic	HR 95%CI	HR 95%CI	
LDL-C strata (mg/dL)			
<55	3.35 (1.83–6.11)	2.69 (1.17–6.17)	**0.019**
55 to 69	2.73 (1.71–4.30)	2.81 (1.65–4.79)	**<0.001**
70 to 99	1.55 (1.09–2.20)	1.49 (0.96–2.32)	0.074
100 to 129 (reference)	1	1	
130 to 159	1.21 (0.82–1.79)	0.96 (0.56–1.64)	0.868
≥160	1.34 (0.85–2.10)	1.68 (0.95–2.95)	0.074
LDL-C < 100 mg/dL			
Overall	1.66 (1.29–2.14)	1.67 (1.21–2.30)	**0.002**
By age			
<60	1.20 (0.81–1.77)	1.18 (0.72–1.94)	0.504
≥60	1.66 (1.18–2.35)	2.12 (1.35–3.34)	**0.001**
By statin use			
Yes	1.67 (1.10–2.53)	1.34 (0.86–2.16)	0.224
No	1.86 (1.27–2.74)	1.81 (1.14–2.86)	**0.011**
By sex			
Men	1.49 (1.10–2.03)	1.49 (0.98–2.25)	0.060
Women	1.82 (1.17–2.85)	2.01 (1.19–3.39)	**0.009**
By cause of death **			
Cancer	1.62 (0.86–3.05)	2.55 (1.10–5.91)	**0.030**
Cardiovascular disease	1.65 (0.79–3.45)	0.75 (0.29–1.93)	0.550
Others ***	1.49 (0.66–3.34)	1.49 (0.66–3.34)	0.339
Excluding deaths in			
first 5 years (*n* = 2104)	1.68 (1.27–2.21)	1.60 (1.19–2.16)	**0.002**
LDL-C < 70 mg/dL			
Overall	2.33 (1.66–3.26)	2.27 (1.51–3.41)	**<0.001**
After excluding deaths			
In the first 5 years (*n* = 2104)	2.18 (1.50–3.19)	2.09 (1.40–3.11)	**<0.001**

* Cox proportional hazards regression for age, sex, race, educational achievement, per capita income, waist–hip ratio, HbA1c, systolic blood pressure, statin use, private health insurance, albumin–creatinine ratio, eGFR, and smoking. ** Cox proportional hazards regression for age, sex, educational achievement, and smoking. *** Blood, neurological, mental, musculoskeletal, chronic kidney, digestive, and respiratory diseases and injuries.

**Table 3:** Crude and adjusted * risks of death according to the achievement of low-density lipoprotein cholesterol (LDL-C) targets for individuals with known diabetes at low CVD risk and without any history of CVD disease now after additional adjustment for frailty assessed at the third clinic visit (follow-up period 2018–2023). ELSA-Brasil.

		Death		
		Crude	Adjusted *	*p*
Characteristic		HR 95%CI	HR 95%CI	
Adjusted for Frailty Phenotype				
	LDL-C <100 mg/dL			
	Overall (*n* = 941)	1.99 (1.20–3.30)	2.05 (1.21–3.47)	**0.008**
	After excluding deaths			
	in first 5 years (*n* = 637)	1.68 (1.27–2.21)	2.01 (1.19–3.41)	**0.009**
	LDL-C <70 mg/dL			
	Overall	1.64 (0.75–3.62)	1.53 (0.69–3.40)	0.298
	After excluding deaths			
	in the first 5 years	2.18 (1.50–3.19)	1.37 (0.55–3.43)	0.504
Adjusted for Frailty Index				
	LDL-C <100 mg/dL			
	Overall (*n* = 1067)	1.88 (1.18–3.00)	1.94 (1.19–3.17)	**0.008**
	After excluding deaths			
	in first 5 years (*n* = 743)	1.68 (1.27–2.21)	1.92 (1.17–3.16)	**0.008**
	LDL-C < 70 mg/dL			
	Overall	1.69 (1.84–3.39)	1.62 (0.80–3.29)	0.181
	After excluding deaths			
	in the first 5 years	2.18 (1.50–3.19)	1.54 (0.67–3.53)	0.308

* Through Cox proportional hazards regression for age, sex, HbA1c, and systolic blood pressure.

## Data Availability

Due to restrictions placed by the ethics committees of the involved institutions, the data used in this study can be made available for research proposals by request to ELSA’s Data Center (estatisticaelsa@gmail.com) and ELSA’s Publications Committee. Additional information can be obtained from the ELSA Coordinator from the Research Center of Rio Grande do Sul (maria.schmidt@ufrgs.br).

## Supplementary Materials

The following supporting information can be downloaded at https://www.mdpi.com/article/10.3390/jcm13247667/s1: Supplementary Figure S1. Flow diagram of selected participants with known diabetes. ELSA-Brasil, 2008–2023. Supplementary Table S1. Criteria used to define the Physical Frailty Phenotype. Supplementary Table S2. Health variables and cut points for the Frailty Index. The index is based on a list of 36 health deficits obtained during the first and third clinic visits and calculated as the sum of deficits present divided by the total of potential deficits for which complete information was available for participants with data on at least 30 health deficits. Supplementary Table S3. Distribution of cause of death among 144 individuals with known diabetes and adjudicated outcomes in ELSA-Brasil between 2008 and 2019. References [17,19,20,21] are cited in the Supplementary Materials.
