# Supplementary material for: On-Target Low-Density Lipoprotein Cholesterol in Adults with Diabetes Not at High Cardiovascular Disease Risk Predicts Greater Mortality, Independent of Early Deaths or Frailty
Source: J Clin Med. 2024 Dec 16;13(24):7667. doi: 10.3390/jcm13247667 (PMC11678450; doi:10.3390/jcm13247667)
Supplement: Supplementary file 1 [file jcm-13-07667-s001.zip › jcm-3296616-supplementary.pdf]

Supplementary Figure S1. Flow diagram of selected participants with known diabetes. ELSA-Brasil, 2008-2023.

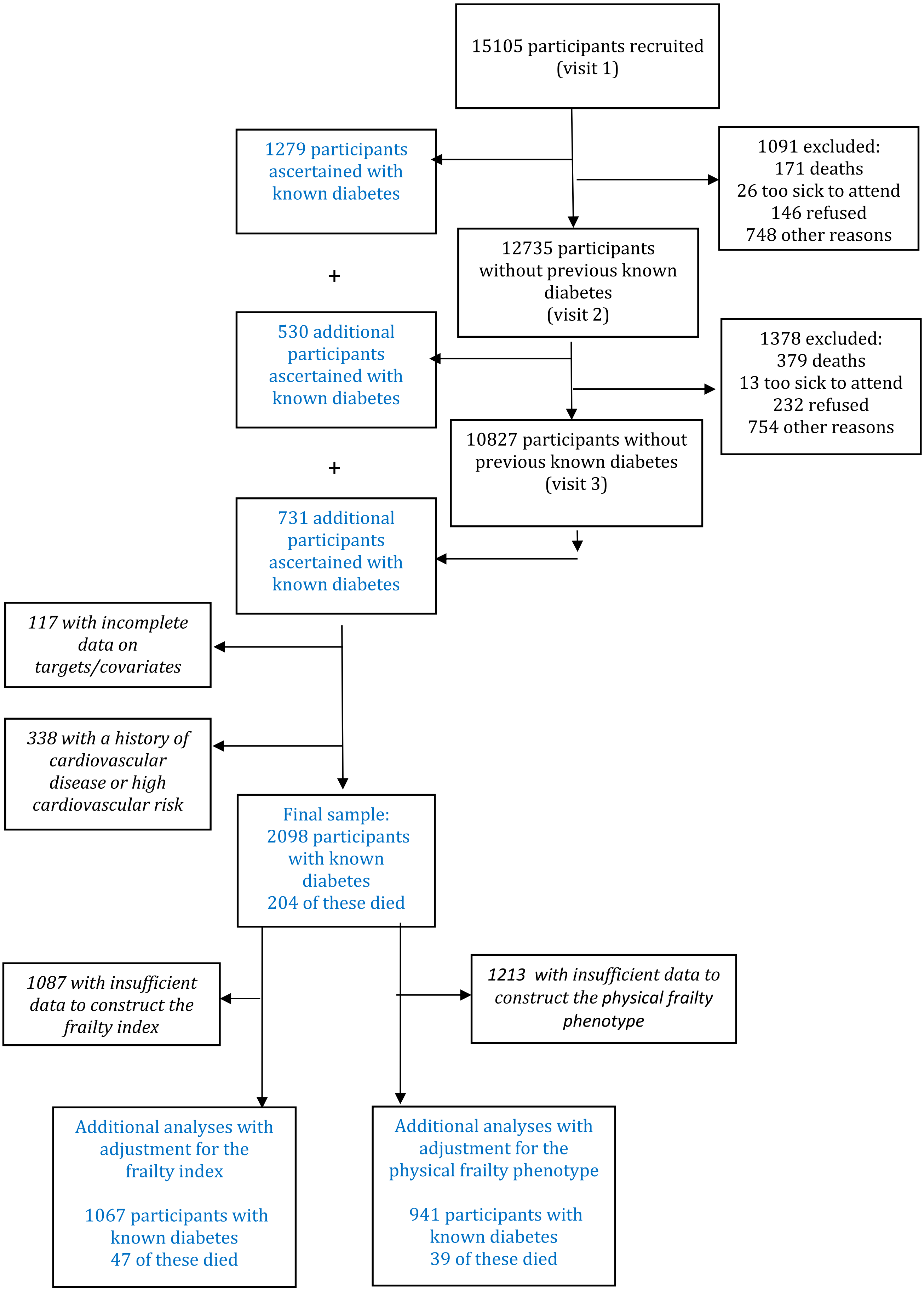

Supplementary Table S1. Criteria used to define the Physical Frailty Phenotype.

Unintentional weight loss

Self-reported loss > 4.5 kg in the previous year without dieting

Exhaustion

Answer “frequently” or “always” to any of the following questions:

“In the last week, how often have you felt that you could not carry on your activities (you started some activity and could not finish it)?”

“In the last week, how often have you felt that everything you did was an effort?”

Low energy expenditure

The number of weekly METs was calculated from the Physical Activity Questionnaire (Craig et al., 2003) and transformed into kcal (METs*weight/60).

Low energy expenditure was defined as < 383 kcal/week for men and < 270 kcal/week for women.

Slow gait speed

Time to walk 15 feet in usual gait (derived from the mean of two measurements of the time to walk 4 meters in usual pace (using assistive devices when needed).

Slowness was defined according to sex and height as follows:

Men:

Height ≤ 173 cm: ≥ 7 s

Height > 173 cm: ≥ 6s

Women:

Height ≤ 159 cm: ≥ 7 s

Height > 159 cm: ≥

Weak grip strength

Handgrip strength (kg) was measured with a Jamar handheld dynamometer and calculated as the mean of two measurements in the dominant hand. Weakness was considered as the lowest quintile according to sex and body mass index quartiles as follows:

Women

BMI ≤ 24 kg/m^2: HG ≤ 20 kg

24 < BMI ≤ 27 kg/m^2: HG ≤ 19 kg

27 < BMI ≤ 30.5 kg/m^2: HG ≤ 19 kg

BMI ≥ 30.5 kg/m^2: HG ≤ 18 kgMen

BMI ≤ 24 kg/m^2: HG ≤ 19 kg

24 < BMI ≤ 27 kg/m^2: HG ≤ 19 kg

27 < BMI ≤ 30 kg/m^2: HG ≤ 20.25 kg

BMI ≥ 30 kg/m^2: HG ≤ 20.25 kg

BMI: body mass index; HG: handgrip strength; MET: Metabolic equivalent of tasks;

Reference:

Craig, C. L., Marshall, A. L., Sjöström, M., Bauman, A. E., Booth, M. L., Ainsworth, B. E., Pratt, M., Ekelund, U., Yngve, A., Sallis, J. F., & Oja, P., 2003. International physical activity questionnaire: 12-country reliability and validity. Med. Sci. Sports Exerc. 35(8), 1381–1395. https://doi.org/10.1249/01.MSS.0000078924.61453.FB

Adapted from: Szlejf C, Suemoto CK, Goulart AC, Santos I de S, Bacchi PS, Fatori D, et al. A pandemic toll in frail older adults: Higher odds of incident and persistent common mental disorders in the ELSA-Brasil COVID-19 mental health cohort. J Affect Disord. 2023 Mar 15;325:392–8.

Supplementary Table S2. Health variables and cut points for the Frailty Index. The index is based on a list of 36 health deficits obtained during the first and third clinic visits and calculated as the sum of deficits present divided by the total of potential deficits for which complete information was available for participants with data on at least 30 health deficits.

List of variables	Deficit definition	Score
BMI[a]	BMI < 18.5 kg/m^2 or BMI ≥ 30 kg/m^2	1
Muscle weakness[a,b]	Muscle weakness was defined as the lowest quintile of HG according to sex and BMI quartiles, as follows:<br><br>Women<br>BMI ≤ 24 kg/m^2: HG ≤ 20 kg<br>24 < BMI ≤ 27 kg/m^2: HG ≤ 19 kg<br>27 < BMI ≤ 30.5 kg/m^2: HG ≤ 19 kg<br>BMI ≥ 30.5 kg/m^2: HG ≤ 18 kg<br><br>Men<br>BMI ≤ 24 kg/m^2: HG ≤ 19 kg<br>24 < BMI ≤ 27 kg/m^2: HG ≤ 19 kg<br>27 < BMI ≤ 30 kg/m^2: HG ≤ 20.25 kg<br>BMI ≥ 30 kg/m^2: HG ≤ 20.25 kg	1
Slow gait speed[a,c]	Slowness was defined as usual gait speed[b] < 0.8 m/s (Cruz-Jentoft et al., 2018)	1
Repeated chair stands[a,d]	12s < Time to complete the task ≤ 15s (Dodds et al, 2021)<br>Time to complete the task > 15s or unable to perform the task without using the arms (Cruz-Jentoft et al., 2018)	0.5<br>1
Weight loss[a]	Self-reported weight loss > 4.5 kg in the last year without dieting	1
Falls[a]	Self-reported history of falls in the last year	1
Difficulty in walking[a]	Answer "very hard" or "I am unable" to the question "How hard is it for you to walk one kilometer?"<br>Answer "very hard" or "I am unable" to the question "How hard is it for you to walk 400 meters?"	0.5<br>1
Difficulty in climbing stairs[a]	Answer "very hard" or "I am unable" to the question "How hard is it for you to climb ten steps of stairs without resting?"	1
Difficulty in bending or kneeling[a]	Answer "very hard" or "I am unable" to the question "How hard is it for you to bend or kneel?"	1
Difficulty in carrying weight[a]	Answer "very hard" or "I am unable" to the question "How hard is it for you to carry weights of up to 5kg, such as a shopping bag?"	1
Difficulty in performing household chores[a]	Answer "very hard" or "I am unable" to the question, "How hard is it for you to perform light household chores (such as making a bed, dusting, and taking out the trash)?"	1
Difficulty in managing own medicines[a]	Answer "very hard" or "I am unable" to the question "How hard is it for you to manage your own medicines (such as following the prescribed timing	1

	and dosage and removing pills from the blister)?”	
Difficulty in managing money[a]	Answer “very hard” or “I am unable” to the question “How hard is it for you to manage your own money (such as controlling your money and using money, credit cards, or cheques for shopping and paying bills)?”	1
Difficulty to prepare a hot meal[a]	Answer “very hard” or “I am unable” to the question “How hard is it for you to prepare a hot mail (such as thinking about the menu, peeling, slicing, mixing, cooking, and serving)?”	1
Cognitive deficit[a]	Mini-mental state examination score according to education level (Brucki et al.,):<br>Up to middle school: < 26.5<br>High school: < 28<br>Undergraduate: < 29	1
Struggle to carry on[a]	Answer to the question, “In the last week, how often have you felt that you could not carry on your activities (you started some activity and could not finish it)?”:<br>“Sometimes”<br>“Frequently” or “Always”	0.5<br>1
Effort[a]	Answer to the question, “In the last week, how often have you felt that everything you did was an effort?”:<br>“Sometimes”<br>“Frequently” or “Always”	0.5<br>1
Hearing impairment[a]	Answer “yes” to the question “Do you feel that you have been losing your hearing?”	1
Self-reported health status[a]	Answer to the question, “Comparing to people the same age, how do you consider your current health state?”:<br>Good<br>Fair<br>Poor<br>Very poor	0.25<br>0.50<br>0.75<br>1
Diabetes[e]	Self-reported use of hypoglycemic agents, fasting blood glucose ≥ 126 mg/dL, 2-hour postprandial glucose test ≥ 200 mg/dL, or glycated hemoglobin ≥ 6.5%	1
Hypertension[e]	Use of anti-hypertensive drug, systolic blood pressure ≥ 140 mmHg, or diastolic blood pressure ≥ 90 mmHg	1
Coronary artery disease[f]	Previous history of myocardial infarction or myocardial revascularization	1
Stroke[f]	Previous history of stroke	1
Heart failure[g]	Self-reported	1
Asma[f]	Self-reported	1
COPD[f]	Self-reported	1
Rheumatic disease[g]	Self-reported	1
Renal disease[g]	Self-reported	1
Thromboembolic disease[g]	Self-reported	1

Hepatic disease[g]	Self-reported	1
Neoplasia[g]	Self-reported	1
Low HDL-cholesterol[a]	HDL-cholesterol < 50 mg/dL for women and < 40 mg/dL for men or use of medication for low HDL-cholesterol	1
Hypertriglyceridemia[a]	Triglycerides ≥ 150 mg/dL or use of medication for hypertriglyceridemia	1
Visual impairment[g]	Self-reported cataract, glaucoma, retinal detachment, retinal degeneration	1
Smoking status[a]	Previous smoker	0.5
	Current smoker	1
Excessive alcohol consumption[a]	Binge drinking or weekly consumption > 21 units for men and > 14 units for women	1

BMI: body mass index; COPD: chronic obstructive pulmonary disease; HDL: high-density lipoprotein; HG: handgrip strength

[a]Assessed at the ELSA-Brasil study's onsite wave 3

[b]Handgrip strength (in kg) measured with a Jamar handheld dynamometer and calculated as the mean of two measurements in the dominant hand.

[c]Usual gait speed (in m/s) was assessed by measuring the time to walk 4 meters in usual pace (with the use of assistive devices when needed). The mean of two different measurements was considered.

[d]Mean of two measurements of the time participants needed to rise five times from a chair without using their arms.

[e]Composite of assessments from the ELSA-Brasil study's onsite waves 1 and 3

[f]Composite of assessments from the ELSA-Brasil study's onsite wave 1

[g]Assessed at the ELSA-Brasil study's onsite wave 1

References:

1- *Brucki, S. M., Nitrini, R., Caramelli, P., Bertolucci, P. H., & Okamoto, I. H., 2003. Suggestions for utilization of the mini-mental state examination in Brazil. Arq. Neuropsiquiatr., 61, 777–781. https://doi.org/10.1590/s0004-282x2003000500014*

2- *Cruz-Jentoft, A. J., Bahat, G., Bauer, J., Boirie, Y., Bruyère, O., Cederholm, T., Cooper, C., Landi, F., Rolland, Y., Sayer, A. A., Schneider, S. M., Sieber, C. C., Topinkova, E., Vandewoude, M., Visser, M., Zamboni, M., & Writing Group for the European Working Group on Sarcopenia in Older People 2 (EWGSOP2), and the Extended Group for EWGSOP2, 2019. Sarcopenia: revised European consensus on definition and diagnosis. Age Ageing. 48, 16–31. https://doi.org/10.1093/ageing/afy169*

3- *Dodds, R. M., Murray, J. C., Granic, A., Hurst, C., Uwimpuhwe, G., Richardson, S., Brayne, C., Matthews, F. E., Sayer, A. A., & MRC CFAS, 2021. Prevalence and factors associated with poor performance in the 5-chair stand test: findings from the Cognitive Function and Ageing Study II and proposed Newcastle protocol for use in the assessment of sarcopenia. J. Cachexia Sarcopenia Muscle. 12, 308–318. https://doi.org/10.1002/jcsm.12660*

**Adapted from Szlejf C, Suemoto CK, Goulart AC, Santos I de S, Bacchi PS, Fatori D, et al. A pandemic toll in frail older adults: Higher odds of the incident and persistent common mental disorders in the ELSA-Brasil COVID-19 mental health cohort. J Affect Disord. 2023 Mar 15;325:392–8.*

Supplementary Table S3. Distribution of cause of death among 144 individuals with known diabetes and adjudicated outcomes in ELSA-Brasil between 2008 and 2019.

Cause	Deaths N (%)
All	144 (100)
Cardiovascular diseases	53 (36.8)
Cancer	48 (33.3)
Digestive diseases	10 (6.9)
Chronic kidney diseases	8 (5.5)
Injuries	7 (4.8)
Chronic respiratory diseases	4 (2.6)
Miscellaneous*	14 (9.0)

**blood, neurological, mental (suicide) and musculoskeletal disease*
